# Mortality Prediction in Hip Fracture Patients: Physician Assessment Versus Prognostic Models

**DOI:** 10.1097/BOT.0000000000002412

**Published:** 2022-05-19

**Authors:** Julian Karres, Ruben Zwiers, Jan-Peter Eerenberg, Bart C. Vrouenraets, Gino M. M. J. Kerkhoffs

**Affiliations:** a Department of Orthopaedic Surgery, Amsterdam UMC, Amsterdam, The Netherlands;; b Department of Surgery, Tergooi Hospital, Hilversum, The Netherlands; and; c Department of Surgery, OLVG Hospital, Amsterdam, The Netherlands.

**Keywords:** hip fracture, mortality, risk prediction, prognostic model, physician judgment

## Abstract

To evaluate 2 prognostic models for mortality after a fracture of the hip, the Nottingham Hip Fracture Score and Hip Fracture Estimator of Mortality Amsterdam and to compare their predictive performance to physician assessment of mortality risk in hip fracture patients.

**Design:**

Prospective cohort study.

**Setting:**

Two level-2 trauma centers located in the Netherlands.

**Patients:**

Two hundred forty-four patients admitted to the Emergency Departments of both hospitals with a fractured hip.

**Intervention:**

Data used in both prediction models were collected at the time of admission for each individual patient, as well as predictions of mortality by treating physicians.

**Main Outcome Measures:**

Predictive performances were evaluated for 30-day, 1-year, and 5-year mortality. Discrimination was assessed with the area under the curve (AUC); calibration with the Hosmer–Lemeshow goodness-of-fit test and calibration plots; clinical usefulness in terms of accuracy, sensitivity, and specificity.

**Results:**

Mortality was 7.4% after 30 days, 22.1% after 1 year, and 59.4% after 5 years. There were no statistically significant differences in discrimination between the prediction methods (AUC 0.73–0.80). The Nottingham Hip Fracture Score demonstrated underfitting for 30-day mortality and failed to identify the majority of high-risk patients (sensitivity 33%). The Hip fracture Estimator of Mortality Amsterdam showed systematic overestimation and overfitting. Physicians were able to identify most high-risk patients for 30-day mortality (sensitivity 78%) but with some overestimation. Both risk models demonstrated a lack of fit when used for 1-year and 5-year mortality predictions.

**Conclusions:**

In this study, prognostic models and physicians demonstrated similar discriminating abilities when predicting mortality in hip fracture patients. Although physicians overestimated mortality, they were better at identifying high-risk patients and at predicting long-term mortality.

**Level of Evidence:**

Prognostic Level II. See Instructions for Authors for a complete description of levels of evidence.

## INTRODUCTION

Hip fractures are a challenge for both patients and health care systems. With ageing populations, the absolute number of hip fractures and their impact on society continues to increase.^1,2^ High mortality and morbidity rates after a fracture of the hip are well documented, with reported 30-day mortality approximately 8% and 1-year mortality of up to 25%.^3–5^ Multiple patient factors are associated with an increased mortality, such as age, gender, cognitive status, and various comorbidities.^6,7^

In recent years, several risk models have been developed for the prediction of mortality following a fracture of the hip. The Nottingham Hip Fracture Score (NHFS)^8^ and the Hip fracture Estimator of Mortality Amsterdam (HEMA)^9^ are prognostic models for 30-day mortality based on preoperative patient characteristics. Although these models were initially designed for of 30-day mortality, the NHFS has been assessed for long-term mortality as well, with several studies reporting accurate prediction of 1-year mortality.^10–12^ A validated risk prediction model might identify patients in need of additional care, guide clinical decision making, and inform patients and caregivers.^13^

Although some risk models show good predictive performance, their usefulness in clinical practice remains to be determined.^14–16^ Moreover, the additional benefit of prognostic models over baseline clinical judgment of mortality risk is unclear. Despite numerous studies on prediction models, clinician intuition is rarely assessed, even though prognostic models seldom outperform physician assessment.^17,18^ Physicians have been reported to be able to predict mortality adequately in patients admitted to the emergency department (ED) and intensive care unit.^19,20^ As of now, no studies report on the value of clinical judgment compared with risk prediction models for mortality in hip fracture patients.

This study aims to evaluate 2 prognostic models for mortality in hip fracture patients and to compare their predictive performance with clinical judgment by the treating physician.

## PATIENTS AND METHODS

This prospective dual-center study included patients admitted with a fractured hip to the EDs of the Tergooi and OLVG West hospitals between June 2014 and October 2015. Both hospitals are level-2 trauma centers and serve a predominantly urban population. Inclusion was completed in the ED at the time of admission. Baseline patient characteristics and variables pertaining to both prognostic models were documented, as well as assessment of mortality risk by the treating physician at the ED.

### NHFS

The NHFS was developed in 2008 and recalibrated by the developers in 2012.^8,21^ It has undergone external validation in several studies, although recalibration has been required to accommodate for geographical differences.^22,23^ The NHFS uses 7 variables to calculate the risk of mortality after hip fracture surgery: age, gender, serum hemoglobin, number of comorbidities, institutionalization, malignancy, and the Abbreviated Mental Test Score (AMTS). The recalibrated NHFS uses the formula 100/{1 + *e* ^[5.012 × (NHFS × 0.481)]^} to predict 30-day mortality after hip fracture surgery.

### Hip Fracture Estimator of Mortality Amsterdam

The HEMA was developed in 2018 and consists of 9 variables: age, in-hospital fracture, malnutrition, myocardial infarction, congestive heart failure, pneumonia, renal failure, malignancy, and serum urea.^9^ Between 0.5 and 2 points are attributed for each variable, the total of HEMA points is used to calculate the predicted 30-day mortality risk as a percentage using the formula 100/[1 + *e* ^(3.823−HEMA)^]. The model divides patients into 3 risk groups based on points: low risk with up to 1 HEMA point, intermediate risk with a maximum of 2 points, and high risk with more than 2 HEMA points.

### Physician Assessment

Mortality prediction by clinicians was performed at admission of the patient from the ED, usually after consultations and advanced testing. The treating physician recorded his or her estimation of 30-day and 1-year mortality risk using a visual analogue scale (VAS). The VAS was a 10-centimeter line on the paper research form with an indication of 0% probability of death on de left end and 100% probability on the right. Treating physicians would mark their predictions on the VAS for both 30-day and 1-year mortality for the individual patient. These predictions were measured and recorded into our database as a number between 0 and 100. Additionally, physician rank and total clinical experience rounded to the nearest 6 months were documented.

Data for both prognostic models and physician assessment were collected prospectively. Necessary information on variables for both models was recorded on the research form by the treating physician. Risk calculation of the NHFS and HEMA was only performed by researchers when adding data to the database, to prevent physicians from knowing predicted probabilities as calculated by the models. A known history of cognitive impairment was used as a substitute for the AMTS when calculating the NHFS.^24^ A contralateral hip fracture in the same patient on a different date was counted as a separate case. Missing preoperative characteristics were not substituted but scored as negative in the final computation of individual risk by the prediction models, as would happen in clinical practice. The primary outcome was 30-day mortality, defined as death within 30 days of admission. Secondary outcomes were 1-year and 5-year mortality. Although the prognostic models were initially designed for the assessment of 30-day mortality risk, several studies found reasonable predictive performance for long-term mortality prediction as well.^10,11,25^

Survival data were verified using hospital records and national databases. Patient or proxy consent was obtained in all cases. This study was approved by the institutional review boards of both hospitals.

### Statistical Analysis

Mortality prediction of prognostic models and physicians was evaluated by performance measures for discrimination, calibration, and clinical usefulness.

Discrimination refers to the ability of a prediction method to distinguish between surviving and dying patients. It was analyzed by means of the area under the receiver operating characteristic curve (AUC).^26^ An AUC of 1.00 demonstrates perfect discrimination; an AUC of 0.50 indicates a complete lack of discriminative ability (ie, prediction by random chance). An AUC of >0.70 was considered sufficiently accurate for mortality prediction.^27^

Calibration describes the agreement between predicted and observed mortality rates. It was evaluated using the Hosmer–Lemeshow goodness-of-fit test, where a significant outcome indicates a lack of fit.^28^ Additionally, calibration was assessed with calibration plots, calibration in the large, and calibration slope.^29,30^ Calibration plots graph predicted probabilities against observed frequencies for grouped cases. Perfect calibration implies that predictions are on a diagonal line and is represented by an intercept (calibration in the large) of 0 and a calibration slope of 1. Calibration in the large is a measure of systematic under- or overestimation; predicted probabilities are systematically underestimated if the intercept is >0 and overestimated if the intercept is <0. Calibration slope quantifies spread and is a measure for over- or underfitting. Overfitting (slope <1) is more common, indicating underestimation of low risks and overestimation of high risks.^31^

Clinical usefulness is the ability of a model to improve the decision-making process.^32,33^ It was assessed in terms of accuracy, sensitivity, and specificity for predictors without a significant lack of fit. A 2 by 2 table was created for each prediction method by dividing patients into 2 groups using a threshold value for predicted probabilities. Threshold values were predicted 30-day mortality >15% (NHFS, HEMA, and physician assessment) and predicted 1-year mortality >50% (physician assessment). Accuracy is the total percentage of patients correctly classified by the prediction method given the threshold value. Sensitivity is the proportion of deceased patients with predicted mortality risk above the threshold value. Specificity is the proportion of surviving patients with predicted mortality risk below the threshold value.

A *P* value of <0.05 was considered statistically significant. Analyses were performed with SPSS Statistics version 26.0 (IBM Corp, NY) and with the val.prob.ci.2 function in R version 3.6.1 (The R Foundation for Statistical Computing, Vienna, Austria).^29^

## RESULTS

In the 17-month period, a total of 244 patients were included after admission to the ED with a fractured hip. Baseline preoperative patient characteristics are described in Table 1. Median age at admission was 83 years, and 163 patients (66.8%) were female. A total of 49 patients (20.2%) had cognitive impairment, and 60 patients (24.6%) were living in an institution before the hip fracture occurred. All patients were treated operatively. Mortality was 7.4% after 30 days, 22.1% after 1 year, and 59.4% after 5 years.

**TABLE 1.** Patient Characteristics, Risk Model Variables and Mortality

Variables		Available Data	Missing Data
Age in years, median (IQR)		83 (74–88)	—
Sex	Female	163 (66.8)	—
	Male	81 (33.2)	
Fracture type	Intracapsular	117 (48.0)	—
	Extracapsular	113 (46.3)	
	Subtrochanteric	14 (5.7)	
Hospital	OLVG West	118 (48.4)	—
	Tergooi	126 (51.6)	
Malignant disease		39 (16.1)	2 (0.8)
Cognitive impairment		49 (20.2)	1 (0.4)
Living in an institution		60 (24.6)	—
Serum haemoglobin in mmol/L, median (IQR)		8.0 (7.2–8.7)	—
Serum urea in mmol/L, median (IQR)		6.9 (5.2–9.0)	17 (7.0)
Serum creatinine in µmol/L, median (IQR)		76 (59–99)	3 (1.2)
30-d mortality		18 (7.4)	—
1-y mortality		54 (22.1)	—
5-y mortality		145 (59.4)	—

Values are n (%) unless otherwise stated.

IQR, interquartile range.

Calculated risk of 30-day mortality was available in all patients for both the NHFS and HEMA (Table 2). Overall predicted 30-day mortality by the NHFS was 7.4%, and individual predictions ranged from 0.7% to 33.6%. The HEMA predicted a 30-day mortality rate of 18.9%, with individual predictions between 2.1% and 99.1%. Physician assessment of mortality risk was available in 243 cases (99.6%). The majority of risk assessments was performed by junior residents (68.6%), and mean clinical experience was 1.5 years. Physicians predicted an overall 30-day mortality of 18.0% and 1-year mortality of 38.2% (Table 2).

**TABLE 2.** Observed and Predicted Mortality Rates and Physician Assessment Data

Variables		Mortality	Range	Missing n (%)
Observed death rate				
30-d mortality		7.4%		—
1-y mortality		22.1%		—
5-y mortality		59.4%		—
Risk model prediction				
NHFS	30-d mortality	7.4%	0.7%–33.6%	—
HEMA	30-d mortality	18.9%	2.1%–99.1%	—
Clinical prediction				
Physician assessment	30-d mortality	18.0%	0%–100%	1 (0.4)
Physician assessment	1-y mortality	38.2%	0%–100%	1 (0.4)
Physician status	n (%)	Experience (years)		
Medical intern	1 (0.4)	0.0	0.0	2 (0.8)
GP in training	21 (8.7)	3.5	1.0–9.0	
Junior resident	166 (68.6)	1.0	0.0–4.0	
Senior resident	54 (22.3)	3.0	0.5–6.0	

GP, general practitioner.

### Discrimination

**FIGURE 1.**
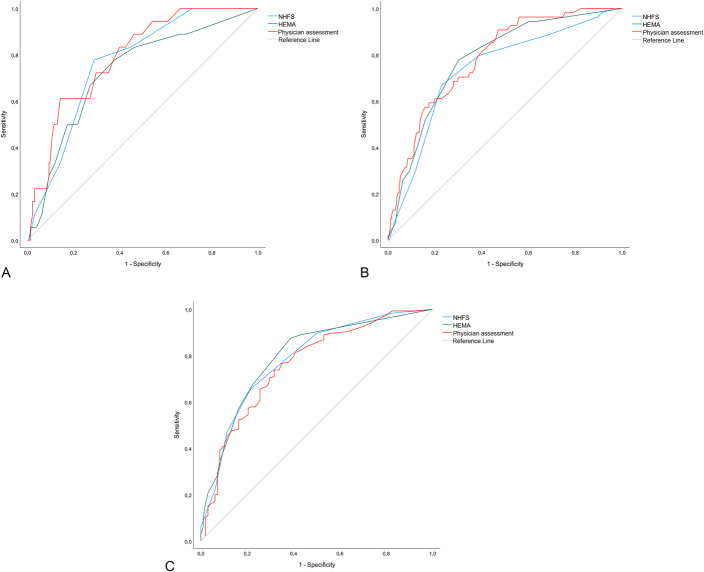
Discrimination plots with receiver operating characteristic curves of prognostic models and physician assessment for (A) 30-day mortality, (B) 1-year mortality, and (C) 5-year mortality. NHFS, Nottingham Hip Fracture Score. A, Receiver operating characteristic curves for 30-day mortality. B, Receiver operating characteristic curves for 1-year mortality. C, Receiver operating characteristic curves for 5-year mortality.

Table 3 describes validation measures for prognostic models and physician assessment. Predictions for 30-day mortality by the NHFS, HEMA, and physician assessment resulted in an AUC of 0.77, 0.73, and 0.79, respectively (Fig. 1A). There were no significant differences in AUC between physician assessment and the NHFS (*P* = 0.7) or the HEMA (*P* = 0.1), or between both prognostic models (*P* = 0.5). Fig. 1 displays the receiver operating characteristic curves of the NHFS, HEMA, and physician assessment for 30-day, 1-year, and 5-year mortality. The AUC for 1-year mortality prediction ranged from 0.74 to 0.79 and the AUC for 5-year mortality ranged from 0.76 to 0.80 (Table 3). As with 30-day mortality, there were no significant differences in AUC between prediction methods for long-term mortality.

**TABLE 3.** Validation Measures for Prognostic Models and Physician Assessment of Mortality in Hip Fracture Patients

Prediction	Discrimination	Calibration	Clinical Usefulness		
	AUC	H-L	Accuracy	Sensitivity	Specificity
30-d mortality					
NHFS	0.77 (0.67–0.86)	0.051	82%	33%	86%
HEMA	0.73 (0.61–0.85)	0.754	73%	67%	73%
Physician assessment	0.79 (0.70–0.88)	0.765	64%	78%	63%
1-y mortality					
NHFS	0.74 (0.66–0.81)	**0.013**	—	—	—
HEMA	0.78 (0.71–0.85)	**0.015**	—	—	—
Physician assessment	0.79 (0.72–0.85)	0.797	71%	65%	73%
5-y mortality					
NHFS	0.78 (0.73–0.84)	**0.001**	—	—	—
HEMA	0.80 (0.74–0.85)	<**0.001**	—	—	—
Physician assessment	0.76 (0.70–0.82)	0.547	—	—	—

Values in parentheses are 95% confidence intervals, bold values are statistically significant.

AUC, area under the curve; H–L, Hosmer–Lemeshow goodness-of-fit test.

Clinical usefulness assessed with threshold value for predicted 30-d mortality >15% and predicted 1-y mortality >50%.

### Calibration

**FIGURE 2.**
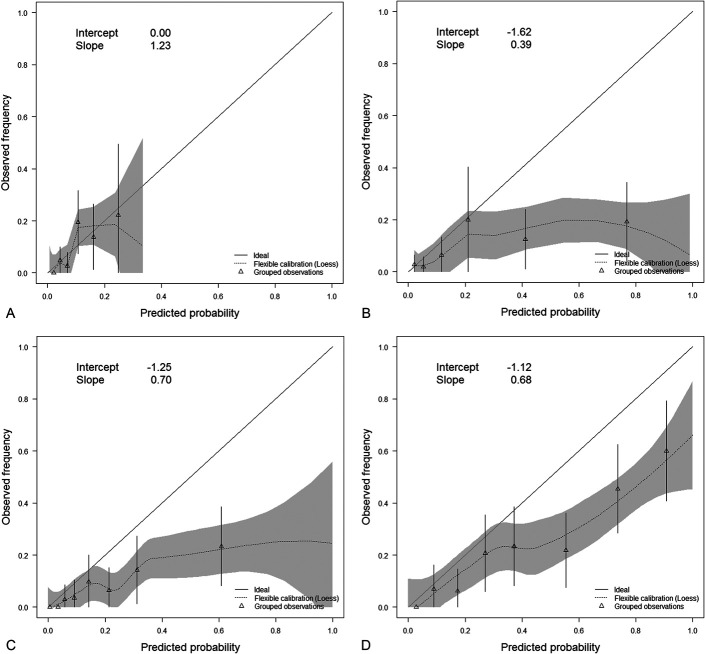
Calibration plots for 30-day mortality prediction by (A) the Nottingham Hip Fracture Score, (B) the Hip Fracture Estimator of Mortality Amsterdam, (C) physician assessment, and (D) 1-year mortality prediction by physician assessment. Triangles represent cases grouped by predicted risk with 95% confidence intervals. The intercept represents calibration-in-the-large and should be as close as possible to 0. The calibration slope should be as close as possible to 1. A, Calibration plot of the NHFS for 30-day mortality. B, Calibration plot of the HEMA for 30-day mortality. C, Calibration plot of physician assessment for 30-day mortality. D, Calibration plot of physician assessment for 1-year mortality.

The Hosmer–Lemeshow goodness-of-fit test demonstrated a significant lack of fit for the NHFS and HEMA for 1-year and 5-year mortality predictions (Table 3). Calibration plots representing the agreement between predicted and observed 30-day mortality rates of the NHFS, and the HEMA are shown in Figs. 2A, B. Calibration in the large of the NHFS was 0.00, the calibration slope was 1.23 indicating underfitting. Calibration in the large of the HEMA was −1.62 with a calibration slope of 0.39, representing both systematic overestimation and severe overfitting. Calibration plots for physician assessment of 30-day and 1-year mortality demonstrated overestimation and some overfitting (Figs. 2C, D). Calibration in the large of physician assessment for 30-day mortality was −1.25, and the calibration slope was 0.70. Calibration in the large of physician assessment for 1-year mortality was −1.12, and the calibration slope was 0.68.

### Clinical Usefulness

Clinical usefulness of both prognostic models and physician assessment is reported in Table 3. With predicted 30-day mortality risk of >15% as threshold value, the accuracy of the NHFS was 82%, with a sensitivity of 33%, and specificity of 86%. Accuracy of the HEMA was 73%, with a sensitivity of 67% and a specificity of 73%. Accuracy of physician assessment was 64%, with a sensitivity of 78% and specificity of 63%. Clinical usefulness outcomes for physician assessment of 1-year mortality with >50% as threshold value are reported in Table 3 as well.

## DISCUSSION

In this study, the discriminative ability of physicians was similar to that of prognostic models for mortality in hip fracture patients. Discrimination was sufficiently adequate for 30-day, 1-year, and 5-year mortality, with no significant difference between the prediction methods. However, there were differences in calibration and clinical usefulness outcomes.

The NHFS had near perfect calibration in the large for 30-day mortality, but the calibration slope demonstrated underfitting (overestimation of low risks and underestimation of high risks). This resulted in a low sensitivity of 33% when assessing clinical usefulness. The limited identification of high-risk patients was demonstrated by the calibration plot as well, in which there were no subgroups with high predicted probabilities (Fig. 2A).

The HEMA demonstrated considerable systematic overestimation of 30-day mortality and substantial overfitting. The combination of underestimated low risks and systematic overestimation resulted in relatively adequate risk predictions up to a predicted probability of death within 30 days of approximately 20% (Fig. 2B). In higher risk predictions, overestimation became extreme. Clinical usefulness for the identification of high-risk patients by the HEMA was moderate with a sensitivity of 67%.

Physician assessment overestimated 30-day mortality but less so than the HEMA. There was some overfitting, and low risk predictions were more accurate than high risk predictions (Fig. 2C). Although accuracy and specificity were lower than in the 2 prognostic models, prediction by physicians resulted in the highest sensitivity; 78% of patients who died within 30 days had a predicted mortality risk of >15%.

Only physicians were somewhat able to predict long-term mortality to some extent. Although physician assessment of 1-year mortality resulted in some overestimation and overfitting, the calibration plot revealed reasonably adequate 1-year mortality predictions even in high-risk patients (Fig. 2D). The NHFS and HEMA both demonstrated a significant lack of fit for 1-year and 5-year mortality prediction. Because both models were developed for 30-day assessment, their use in long-term mortality prediction might be limited.^8,9^ Although several studies have reported reasonable accuracy of the NHFS for 1-year mortality, this study could not corroborate these findings.^10–12^

Numerous prognostic models have been developed for mortality prediction in hip fracture patients, and new models are proposed every year.^11,34–38^ The NHFS is the most studied model and demonstrated adequate discrimination for 30-day and 1-year mortality in different studies, although recalibration has occasionally been necessary.^12,16,23,25,39^ Despite multiple validation studies, there are no reports on its calibration slope nor clinical usefulness, leaving underfitting or lack of sensitivity potentially underreported.^31^

The HEMA prediction model has been developed by us more recently and has not yet undergone external validation.^9^ Overfitting as seen in this study is a common problem in prediction models, often arising from development with a small number of events.^26,29^ Factors used by the HEMA such as in-hospital fracture or pneumonia are infrequent and might be less suitable for use in large-scale clinical risk prediction models.

Physician assessment was superior to risk prediction models when identifying high-risk patients in this study and better at long-term mortality prediction. Research comparing the value of clinical intuition to prognostic models is limited. Research by Schriger et al^17^ found that physician judgment was assessed in only 15% of prospective studies evaluating clinical decision aids. Furthermore, prognostic models only outperformed physician assessment in 2 of the 21 studies that reported on physician judgment.^17^

Several studies have investigated physician assessment of mortality risk in patients admitted to the ED. Herzog et al^40^ reported accurate physician prediction of mortality in patients admitted with generalized weakness and fatigue. Similarly, Zelis et al^20^ found that clinical intuition documented at admission could predict mortality and other adverse outcomes in older patients. Multiple studies on ED patients have demonstrated an association between physician judgment and increased hospitalization, morbidity, and mortality.^41–43^

To our knowledge, this is the first study comparing physician assessment with prognostic models for mortality in hip fracture patients. Strengths of this study include the prospective collection of data and long-term follow-up. Furthermore, analysis of predictive performance was extensive, including assessment of calibration plots and clinical usefulness measures, often underreported in validation studies of prediction models.^26,31^

Limitations of this study include its relatively small sample size and unavailable data for the AMTS for calculating the NHFS. Cognitive impairment is an independent predictor of mortality after hip fracture surgery and was used to substitute for the AMTS, as suggested by other investigators.^24,44,45^ Although prognostic models are not routinely used for hip fracture patients in our hospitals, familiarity with their components such as relevant comorbidities might influence a physician's assessment of mortality. To minimize this influence, the calculated risk predictions were unavailable to physicians at the time of their prediction. Finally, predictions were often done by residents working in the ED. These residents would be the physicians that admit patients after the necessary tests and consults, provide perioperative care and talk to patients and their families. Although their clinical experience might be limited, these physicians provide complete patient assessment and use of their risk prediction is reflective of daily clinical practice. It is unclear whether the accuracy of physician assessment would improve if predictions were done by more experienced clinicians such as attending surgeons.^46,47^

Mortality prediction in hip fracture patients is complicated, and the perfect prognostic model does not exist. Current models might allow for risk stratification when comparing patient groups (eg, between hospitals), but their capability for individual risk prediction is limited. Adequate risk assessment of individual patients and improvement on clinical judgment are however essential for a model to be clinically helpful and improve standard of care. Accurate mortality prediction could aid patients, their families, and caregivers in the decision-making process surrounding hip fracture care. It might even guide complicated discussions regarding decisions on whether to operate. As of now, these prognostic models fail to play such a substantial role in clinical decision making.^16^

In this study, prediction models could not outperform physicians when assessing mortality risk and did not improve on baseline clinical judgment. Physicians demonstrated moderate discriminative abilities similar to those of prognostic models. Moreover, physicians seemed better at identifying high-risk patients, arguably the most important factor influencing clinical decision making. When counselling hip fracture patients and their families about prognosis, or when deciding on surgical or palliative strategies, physician assessment might be more valuable than these models. Future studies should include clinical judgment when investigating mortality prediction in hip fracture patients.
